# On solution existence of MHD Casson nanofluid transportation across an extending cylinder through porous media and evaluation of priori bounds

**DOI:** 10.1038/s41598-021-86953-1

**Published:** 2021-04-08

**Authors:** Sohaib Abdal, Sajjad Hussain, Imran Siddique, Ali Ahmadian, Massimiliano Ferrara

**Affiliations:** 1 School of Mathematics, Northwest University, No.229 North Taibai Avenue, Xi’an, 7100069 China; 2 School of Aerospace and Mechanical Engineering, Nanyang Technological University, Singapore, Singapore; 3 Department of Mathematics, University of Management and Technology, Lahore, Pakistan; 4 Institute of IR 4.0, The National University of Malaysia, UKM, 43400 Bangi Selangor, Malaysia; 5 Department of Law, Economics and Human Sciences & Decisions Lab, University Mediterranea of Reggio Calabria, Reggio Calabria, Italy

**Keywords:** Metamaterials, Nanoparticles, Mathematics and computing

## Abstract

It is a theoretical exportation for mass transpiration and thermal transportation of Casson nanofluid over an extending cylindrical surface. The Stagnation point flow through porous matrix is influenced by magnetic field of uniform strength. Appropriate similarity functions are availed to yield the transmuted system of leading differential equations. Existence for the solution of momentum equation is proved for various values of Casson parameter $\beta$, magnetic parameter *M*, porosity parameter $K_p$ and Reynolds number *Re* in two situations of mass transpiration (suction/injuction). The core interest for this study aroused to address some analytical aspects. Therefore, existence of solution is proved and uniqueness of this results is discussed with evaluation of bounds for existence of solution. Results for skin friction factor are established to attain accuracy for large injection values. Thermal and concentration profiles are delineated numerically by applying Runge-Kutta method and shooting technique. The flow speed retards against *M*, $\beta$ and $K_p$ for both situations of mass injection and suction. The thermal boundary layer improves with Brownian and thermopherotic diffusions.

## Introduction

Non Newtonian fluids do not satisfy the Newton’s law of viscosity e.g. juice of apple, fuel oils, cream, honey, blood, toothpaste etc. Casson fluid is a prominent type of fluid among all of them. It is claimed that for some fluids this rheological model is better as compared to the viscoelastic model. This model is suitable for blood as well as for chocolate rheology. Basically, the sample of casson fluids is made up due to the connections or interactions between the phases of liquids and solid. When yield stress becomes compulsory and it is lower than the shear stress, Cason fluids behaves like solids. e.g. Soup, tomato, honey, etc. Human blood is also an example of Casson fluid. Shah et al.^1^ investigated the flow of Casson nano fluid along with activation energy as well as the chemical reaction by using the stretched surface. Oyelakin et al.^2^ studied gyrotactic micro-organism in Casson nano-fluid flow. Reza et al.^3^ utilized finite difference analysis on unsteady MHD flow of Casson fluid. Effect of slip boundary conditions of time dependent Casson nano-fluid passing over sheet were discussed by Oyelakin et al.^4,5^. Mondal et al.^6^ discussed three dimensional casson nano-fluid over a porous stretch sheet. Non-Newtonian fluid together with various geometries are studied in^7–10^.

Some analytical uses of straight-line flows along with the stretching/shrinking sheet or by the regular string consist in different processing of collecting i.e. industry of polymer, a porous stretching/shrinking of plastic films, artificial filaments, fibers of counterfeit, melting of metals, expulsion of metals, persistent throwing, glass blowing etc.^11^. Firstly, the problem of the stretching sheet was discussed by Sakiadis^12,13^. Awaludin et.al.^14^ discussed the boundary layer flow of magnetohydrodynamic over stretching and shrinking sheet. Dzulkifli et. al.^15^ analyzed the flow of stagnation point as well as relocation of heat over stretching and stretching sheet by using the nano fluid along with the impact of slip velocity. Bakar et.al.^16^ disussed on analysis of relocation of heat along with the nanofluid by using stretching / shrinking surface with the impact of suction. Malvandi et al.^17^ discussed about the flow of stagnation point by using the nonlinear stretching/shrinking sheet which is a porous surface.

In 1942, Haanas Alfren introduced terminology of MHD “Magnetohydrodynamic”. Large number scholars has done researches to understand the properties of MHD and to check these properties impact with various terms of nanofluid Now a days using in various fields of life such as astrophysics, medical science, geography, and many other. Impact of activation energy of Arrhenius over a nonlinear stretching surface with convective third grade nanofluid in MHD flow investigated by Hayat et al.^18^. Nanomaterials treatment regardless of the imposition of MHD streamline considering the melting sheet reviewed by Dinh et al.^19^. Explored MHD nanofluid flow over a porous formation of shrinking walls of entropy conducted by Rashid et al.^20^. Research taken on magnetohydrodynamic current of nanofluid through a vertical permeable plate that flows semi-infinitely by Pavar et al.^21^. Oyelakin et al.^22^ discussed MHD flow of tangent hyperbolic. Chen et al.^23^ studied Mixed convection nanofluid stream in vertical channel entropy production in magnetohydrodynamic. Arifuzzaman et al.^24^ studied heat and mass transfer analysis of MHD through a porous plate.

Nano liquids are potential heat exchange fluids with improved thermos-physical properties and heat trade execution can be associated with various tools for better exhibitions Work nowadays in the area of nano-materials grown rapidly due to its comprehensive implementations in variety of fields. Scholars paid so interest in recent array in this field due to their various applications, heat and mass transfer^25^, in the engineering and industrial appliances sector, for example Nuclear reactor cooling, furnace, coolant, polymer Process, filament plastics. Improving fluid thermal conductivity of nanoparticles studied by Choi and Eastman^26^. Nanofluid jet cooling fluid flow and heat transfer analysis on a hot surface with varying roughness studded by Mahdavi et al.^27^. Oyelakin et al.^28^ discussed non-linear radiation in Casson nano-fluid flow. Bagh et al.^29^ discussed time dependent water based nano fluid on an extending sheet. Ali et al.^30^ studied the impact of Stefan blowing for nanofluid flow. Three dimensional casson-carreau nanofluid flow numerical scrutinization interrogated by Shahid et al.^31^. Reza et al.^32^ studied multiphase behavior of fluid flow with nanoparticles. Sadeghi et al.^33^ studied ferro-fluid with the presence of two cylinders. Seyyedi et al.^34^ solved different shape nano-particles by using entropy generation. Dogonchi et al.^35^ investigated heat and mass transfer effects of nanofluid in an irregular triangular enclosure. Similar work were done by^36–40^.

A glance of the related studies of flow across a cylindrical surface is mostly treated with implementation of numerical methods. Mastroberardino and Siddique^41^ presented numerical solution for MHD flow of Newtonian fluid towards a stretching cylinder. They discussed the conditions for existence and uniqueness of the solution. Motivated from this rarely considered work we studied the fluid flow through porous medium in the existence of applied magnetic field. To our interest we proved the existence and uniqueness of this extended fluid flow problem. we also evaluated the priori bounds for skin friction factor, As for as we know these aspects for the flow of Casson fluids are never explained in the existing studies. The innovation of the work highlighted the existence of solution with uniqueness of results and bounds for skin friction. Moreover, numerical solution of this work is obtained by employing shooting base numerical method codded in matlab script. This exploration may find application in blood rheology, food processing and metallurgy.

## Mathematical analysis

In this segment, we are concerned with the following incompressible Casson nanofluid model^42^

1 $$\begin{aligned} {\left. \begin{aligned} \partial _t \rho + u.\nabla \rho = 0\\ \rho (\partial _t u + u.\nabla u) = - \nabla \rho - (1+\frac{1}{\beta })\mu \Delta u - f\\ div \ u = 0\\ \partial _t T + div(u \ T) = \frac{k}{\rho C_p} \nabla ^2 T + \tau (D_B\ \partial _r T \ \partial _r C + \frac{D_T}{T_\infty }(\partial _r T)^2) \\ \partial _t C + div(u \ C) = D_B \ \nabla ^2 C + + \frac{D_T}{T_\infty } (\partial _r T)^2 \\ \end{aligned}\right\} } \end{aligned}$$

**Figure 1:**
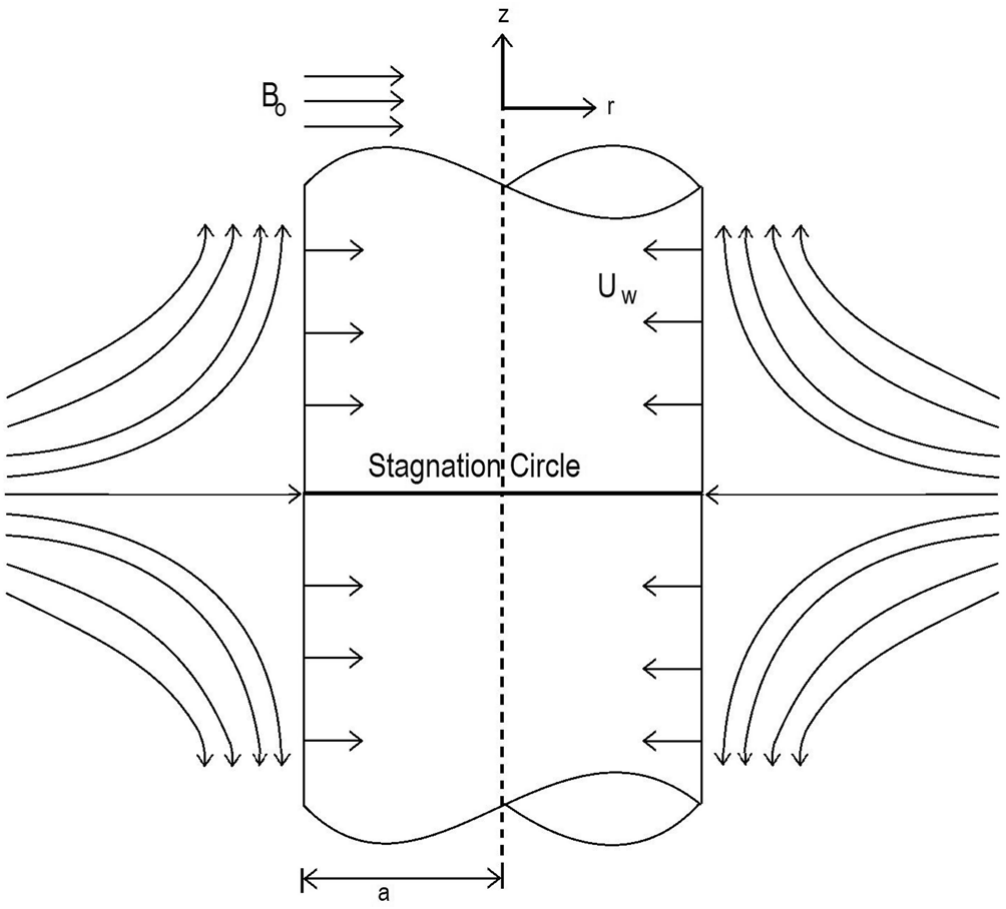
Physical configuration and coordinate system.

Consider an incompressible and electrically conducting Casson fluid which flows steady state across an axially extending cylinder of radius = *a*. Velocity of the stretching wall of the cylinder is $U_w = ca\gamma$. The mass suction across the wall is $w_w = 2cz$, Here *c* is strain rate of the radial flow and $\gamma$ is permeability parameter. The fluid flows through a porous medium of Darcy resistance. There is a non varying magnetic field of intensity $B_o$ that acts normally to the axis of symmetry (see Fig. 1). The temperature $T_w$ and concentration $C_w$ are taken at the cylinder and $T_\infty$ and $C_\infty$ are the far field temperature and concentration. Casson fluid parameter is $\beta$ and $k'$ is the porosity of medium. The formulation in $(r, \theta , z)$ is constituted keeping in view with the assumptions as mentioned above.

2 $$\begin{aligned} {\left. \begin{aligned} \frac{\partial (rw)}{\partial z}+\frac{\partial (ru)}{\partial r}=0,\\ w \frac{\partial w}{\partial z}+u \frac{\partial w}{\partial r} = \nu (1+\frac{1}{\beta })(\frac{\partial ^2 w}{\partial r^2} + \frac{1}{r} \frac{\partial w}{\partial r})-\frac{\sigma B_{0}^2w}{\rho } - \frac{\nu }{k'}w,\\ w \frac{\partial T}{\partial z}+u \frac{\partial T}{\partial r}=\alpha (\frac{\partial ^2 T}{\partial r^2}+\frac{1}{r} \frac{\partial T}{\partial r})+\tau (D_B\frac{\partial T}{\partial r}\frac{\partial C}{\partial r}+ (\frac{\partial T}{\partial y})^2 \frac{D_T}{T_\infty }),\\ w \frac{\partial C}{\partial z}+ u \frac{\partial C}{\partial y}= D_B (\frac{\partial ^2 C}{\partial r^2}+\frac{1}{r} \frac{\partial C}{\partial r}) + \frac{D_T}{T_\infty }\frac{1}{r}(\frac{\partial }{\partial r}(r\frac{\partial T}{\partial r})).\\ \end{aligned}\right\} } \end{aligned}$$

with boundary conditions:

3 $$\begin{aligned} {\left. \begin{aligned} u = U_w,\ w = w_w,\ T = T_w, \ C = C_w,\ at\ r=a,\\ w\rightarrow 0,\ T\rightarrow T_\infty ,\ C \rightarrow C_\infty ,\ as\ r \rightarrow \infty . \end{aligned}\right\} } \end{aligned}$$

In order to yield dimensionless form, similarity transformations are entitled as:

4 $$\begin{aligned} {\left. \begin{aligned} \xi = (\frac{r}{a})^2, \ u = -ca \frac{f(\xi )}{\sqrt{\xi }}, \ w = 2cf'(\xi )z,\ \theta (\xi )-\frac{T-T_\infty }{T_w - T_\infty }=0,\ \phi (\xi )-\frac{C-C_\infty }{C_w - C_\infty }=0. \end{aligned}\right\} } \end{aligned}$$

The first expression in (2) becomes an identity and the remaining’s take the form as follows:

5 $$\begin{aligned} (1+\frac{1}{\beta })\xi f''' + f'' - Re[f'^2-ff'']-(M+K_p)f' = 0 \end{aligned}$$

6 $$\begin{aligned} \xi \theta '' + (1+Pr Re f)\theta ' + \xi Pr [Nb \theta ' \phi ' + Nt \theta '^2] = 0 \end{aligned}$$

7 $$\begin{aligned} \xi \phi '' + (1+Le Re f)\phi ' + \frac{Nt}{Nb}[\xi \theta '' + \theta '] = 0 \end{aligned}$$

Where the expression (3) are transformed:

8 $$\begin{aligned} {\left. \begin{aligned} f(1) = \gamma ,\ f'(1) = 1,\ \theta (1) = 1, \ \phi (1) = 1,\\ f'(\infty )\rightarrow 0,\ \theta (\infty )\rightarrow 0,\ \phi (\infty )\rightarrow 0, \end{aligned}\right\} } \end{aligned}$$

Where $M = \frac{\sigma B_0^2 a^2}{4\nu \rho }$ is magnetic parameter, $Re = \frac{ca^2}{2\nu }$ is Reynolds number, $Pr = \frac{\nu }{\alpha }$ is Prandtl number, $Nb = \frac{\tau D_B(C_w-C_\infty )}{\nu }$ is Brownian motion, $Nt = \frac{\tau D_T(T_w-T_\infty )}{T_\infty \nu }$ is thermophoresis parameter, $Le = \frac{\nu }{D_B}$ represents Lewis number. The physical quantities of interest are $Cf_x$ (skin friction coefficient), $Nu_x$ (local Nusselt number) and $Sh_x$ (local Sherwood number):

$$Cf_x = \frac{\tau _w}{\rho U^2w}$$, $$Nu_x = \frac{aq_w}{k(T_w - T_\infty )}$$, $$Sh_x = \frac{xq_m}{D_B(C_w - C_\infty )}$$,

where $\tau _w$, $q_w$ and $q_m$ denotes shear stress, surface heat flux and surface mass flux,

$$\tau _w = \mu \frac{\partial w}{\partial r}$$, $$q_w = -K \frac{\partial T}{\partial r}$$, $$q_m = -D_B \frac{\partial C}{\partial r}$$ at $$r = a$$,

On solving these quantities with the help of given similarity transformation, we obtain:

$$C_f(Re_x)^{-1/2} = (f''(1)$$, $$Nu_x(Re_x)^{-1/2} = -2\theta '(1)$$, $$Sh_x(Re_x)^{-1/2} = -2\phi '(1)$$,

where, $(Re_x) = \frac{x U_w}{\nu }$ is the local Reynolds number.

## Existence

Consider the BVP (boundary value problem)

9 $$\begin{aligned} (1+\frac{1}{\beta })\xi f''' + f'' - Re[f'^2-ff'']-(M+K_p)f' = 0 \end{aligned}$$

with

$$\begin{aligned} f(1) = \gamma ,\ f'(1) = 1, f'(\infty )\rightarrow 0. \end{aligned}$$

In order to get the corresponding IVP (initial value problem), the missing initial condition is assumed to be

10 $$\begin{aligned} f''(1) = \epsilon , \end{aligned}$$

Here $\epsilon$, is a free parameter is relevant to skin friction parameter and $f(\xi ;\epsilon )$ denotes the solution. It is because an IVP can be uniquely solved (locally). Thus, a topological shooting argument for some choice of $\epsilon$. For convince, the dependence of f on $\epsilon$ may be skipped for some time. The existence of $f'(\xi ;\epsilon )$ for all $\xi > 1$ to satisfy Eq. (8). It may yield a solution to BVP. Two sets X and Y are taken as:

$$\begin{aligned} X = {\epsilon < 0 | \ a\ first\ point\ \xi _X> 1\ is \ such\ that\ f'(\xi ) > 0 \ and\ f''(\xi _X) = 0 \ on\ [1, \xi _X]} \end{aligned}$$

and

$$\begin{aligned} Y = {\epsilon< 0 | \ a\ first\ point\ \xi _Y > 1\ such\ that\ f'(\xi ) < 0 \ and\ f''(\xi _Y) = 0 \ on\ [1, \xi _Y]} \end{aligned}$$

Both of these sets are shown to be open and non-empty in the two Lemmas below:

### Lemma 1

*The set X is non-empty and open.*

### Proof

From Eqs. (9) and (10), for $\xi = 1$,

11 $$\begin{aligned} (1+\frac{1}{\beta })f'''(1) = Re-\epsilon [(1+\frac{1}{\beta }) + R \gamma ] + (M+k_p) \end{aligned}$$

When $\epsilon = 0$, it implies that $f'''(1) = Re > 0$. Then initially $f' > 1$ and $f'' > 0$ on $(1, 1+\delta ]$ for some $\delta > 0$. The continuity of the solutions of IVP and for $\epsilon < 0$ approaching zero, $f'(\xi ;\epsilon )$ approaches $f'(\xi ;0)$, i.e., $f'(\xi ;\epsilon ) > 0$ on $(1, 1+\delta ]$ with $f(1+\delta ;\epsilon ) > 1$. But $f'(\xi ;\epsilon ) < 1$ and non-increasing for $\xi \in (1, 1+\delta _1)$ for some $0< \delta _1 < \delta$. $f'$ is to have a minimum if it is to go over 1. So the existence of first point $\xi _X$ such that $f''(\xi _X;\epsilon ) = 0$ and $f'(\xi _X;\epsilon ) > 0$ on $[1, \xi _X]$. Therefore in case of $\epsilon < 0$ approaching to 0 this implies that $\epsilon$ belong to A. In order to show that X is open, let ${{\bar{\epsilon }}} \in X$ is open, let ${{\bar{\in }}} X$. It is to show that all $\epsilon$ approaching ${{\bar{\epsilon }}}$ are in X. Then $f''(\xi _X) = 0$ and $0< f'(\xi _X) < 1$. At $\xi _X({{\bar{\epsilon }}})$, the Eq. (5) yields

$$\begin{aligned} f'''(\xi _X) = \frac{1}{(1+\frac{1}{\beta })\xi _X}[Re f'^2(\xi _X) + (M+K_p)f'(\xi _X)] > 0. \end{aligned}$$

As the situation for IVP is continuous in its initial conditions, $\epsilon$ is approaching close to ${{\bar{\epsilon }}}$, $f''(\xi ;\epsilon )$ has a root $\xi X(\epsilon )$, near $\xi X({{\bar{\epsilon }}})$ with $f'(\xi ;\epsilon ) > 0$. Thus $\epsilon \in X$. We are there with the only possibility that $f'' = 0$ and $f' = 0$ simultaneously. When these values are substituted in Eq. (5), then $f''' = 0$ to imply $f'(\xi ) = 0$ for all $\xi$. This is a contradiction to Eq. (8). $\square$

### Lemma 2

*The set Y is open and non-empty.*

### Proof

Equation (5) after integration yields as:

12 $$\begin{aligned} (1+\frac{1}{\beta })\xi f''(\xi ) = (1+\frac{1}{\beta })\epsilon + Re \int _{1}^{\xi }(f'^2(z)-f(z)f''(z))dz + (M+K_p)(f(\xi )-\gamma ) \end{aligned}$$

and a subsequent integration by parts yields

13 $$\begin{aligned} (1+\frac{1}{\beta })\xi f''(\xi ) = (1+\frac{1}{\beta })\epsilon + 2Re \int _{1}^{\xi }f'^2(z)dz + Re [\gamma - f(\xi ) f'(\xi ) + (M+K_p)(f(\xi )-\gamma )] \end{aligned}$$

It is to show that there is $\epsilon < 0$, such that $f'$ is equated to zero in the interval (1,2], say, before $f'' = 0$ in strict. Suppose this assertion is not true and consider.

Case (1A). Taking $f'' < 0$, $0< f' < 1$ for $\xi \in (1,2]$, when $\gamma \ge 0$: By integrating $0< f' < 1$ yields $\gamma< f < \gamma +\xi - 1$ on (1,2]. Then Eq. (13), provides:

$$\begin{aligned} f'' < [\frac{\epsilon }{2} + 2Re + Re \gamma + (M + k_p)](1+\frac{1}{\beta }) \end{aligned}$$

By selecting $\epsilon < -2(M+K_p)-2Re\gamma -4Re-2$ to have $f'' < -1(1+\frac{1}{\beta })$ on (1,2] and thus $f'(2) < 0$ which contradicts $f' > 0$ on (1,2].

Case (1B). $f'' < 0$, $0< f' < 1$ for $\xi \in (1,2]$, $\gamma < 0$. Also, the integration of $0< f' < 1$ on (1,2] yields $\gamma< f < \gamma +\xi - 1$ on (1,2]. By employing these conditions in Eq. (13) to get

$$\begin{aligned} f'' < [\frac{\epsilon }{2} + 2Re + Re \gamma + (M + k_p)](1+\frac{1}{\beta }) \end{aligned}$$

Choosing $\epsilon < -2(M+K_p)-4Re-2$ then $f'' < -1(1+\frac{1}{\beta })$ on (1,2] and $f'(2) < 0$, it is a contradiction to $f' > 0$ on (1,2].

Case (2). If there is first point $\xi _1 \in (1,2]$ when $f''(\xi _1) = 0$ with $f'' < 0$ on $(1,\xi _1)$. By taking conditions on $f''$ as in case (1), it results in

$$\begin{aligned} {\left. \begin{aligned} f''< [2Re+\frac{\epsilon }{2}], when \gamma< 0,\\ f'' < [Re \gamma +2Re+\frac{\epsilon }{2}], when \gamma \ge 0. \end{aligned}\right\} } \end{aligned}$$

for $$\xi \in (1,\xi _1]$$. Choosing

$$\begin{aligned} {\left. \begin{aligned} f''< [ -4Re, when \gamma< 0,\\ f'' < -(2Re \gamma +4Re), when \gamma \ge 0. \end{aligned}\right\} } \end{aligned}$$

implies that $f''(\xi _1) < 0$ it contradicts $f''(\xi _1) = 0$.

Case (3). We are left with options that $f'' = 0$ and $f' = 0$, but the process of Lemma 1, yields that $f' \equiv 0$ to contradict Eq. (8).

Hence Y is non void. Now it is to see that Y is open, let ${{\bar{\epsilon }}} \in Y$ with existence of $\xi _Y ({{\bar{\epsilon }}})$ such that $f''\xi _Y ({{\bar{\epsilon }}}) < 0$ and $f'\xi _Y ({{\bar{\epsilon }}}) = 0$. The continuity of the solution of IVP, for $\epsilon$ close to ${{\bar{\epsilon }}}$, there exist $\xi _Y (\epsilon )$ with $f''\xi _Y (\epsilon ) < 0$ and $f'\xi _Y (\epsilon ) = 0$, and so, Y is open.

Thus X and Y are non empty, disjoint and open sets, but $(-\infty ,0)$ is connected and so $X U Y \ne (-\infty ,0)$. Thus, there is $\epsilon ^*$ such that $\epsilon ^*\not \in X$ and $\epsilon ^*\not \in Y$. As already noticed it in not possible to have $f' = 0$ and $f'' = 0$ simultaneously; thus, thus only choice is $f''(\xi ;\epsilon ^*) < 0$ and $f'(\xi ;\epsilon ^*) > 0$ for all $\xi > 1$.

Since $f'$ is bounded below and decreasing, $f'(\infty ;\epsilon ^*) = Z$ exists where $0 \le Z < 1$. It is to see that $Z = 0$. We let $0 \le Z < 1$. As $f'' < 0$ for $\xi > 1$, $f'$ is bounded below by $Z > 0$, and so, f approaches to positive infinity. Finally the term $ff''$ is negative. Equation (5) provides as below:

$$\begin{aligned} \xi f'''(\xi ) = \left[ -f''(\xi ) - (1+\frac{1}{\beta })^{-1}\left[ Re(ff''-f'^2)+(M+K_p)f'(\xi )\right] \right]> ReC^2 = K > 0 \end{aligned}$$

for $\xi$ to be large enough, there exists a point $\xi _2 > 1$ and $\xi > \xi _2$ to imply that

$$\begin{aligned} \xi f'''(\xi ) > \frac{K}{2} \end{aligned}$$

By integrating the above expression

$$\begin{aligned} f''(\xi )> f''(\xi _2) + \frac{K}{2}[ln\ \xi - ln\ \xi _2]\ for\ \xi >\ \xi _2, \end{aligned}$$

Let $\xi \rightarrow \infty$ then $f'' \rightarrow \infty$, it contradicts to the fact that $f'' < 0$. Hence we have $f'(\infty ;\epsilon ^*) = 0$ the following theorem is established. $\square$

### Theorem 1

*There exists a solution to the boundary value problem for any* $Re > 0$ *and* $-\infty< \gamma < \infty$, *to satisfy* $f'(\xi ) > 0$ *and* $f''(\xi ) < 0$ *for all* $\xi > 1$.

## Uniqueness

Now, we prove uniqueness of results:

### Theorem 2

*If* $-\infty< \gamma < \infty$ *and* $Re > 0$, *then we cannot have two solutions for BVP (see* 8*)*

$$\begin{aligned} (1+\frac{1}{\beta })\xi f'''(\xi )+ f''(\xi ) - Re(f'^2-ff'')-(M+K_p)f'(\xi ) = 0 \end{aligned}$$

*when* $$f'(\xi ) > 0$$.

### Proof

From Eq. (5), $f'(\xi ;\epsilon ^*)$ cannot attain maximum. Thus for a solution with $f'(\xi ;\epsilon ^*) > 0$, $f''(\xi ;\epsilon ^*) < 0$. So for any positive solution $0< f'(\xi ;\epsilon ^*) < 1$. Let $v = \frac{\partial f}{\partial \alpha }$. The differentiation of Eq. (5) with respect to $\xi$ yields:

14 $$\begin{aligned} (1+\frac{1}{\beta })\xi f^{iv} + f''' - Re[2f'f'' - ff''' - f'f'']-(M+K_p)f'' = 0 \end{aligned}$$

15 $$\begin{aligned} (1+\frac{1}{\beta })\xi v''' + v'' - Re[2f'v' - vf'' - fv'']-(M+K_p)v' = 0 \end{aligned}$$

associated with

16 $$\begin{aligned} v(1) = v'(1) = 0, v''(1) = 1. \end{aligned}$$

Thus for $\xi > 1$, we have $v'$ positive and increasing and $v' > 0$ and increasing for $\xi >1$.

It is to show a positive maximum does not exists for $v'(\xi ,\epsilon ^*)$. Let a maximum exists at first point for which $v > 0$, $v' > 0$, $v'' = 0$ and $v''' \le 0$. Substituting $v'' = 0$ into Eq. (15) yields

17 $$\begin{aligned} (1+\frac{1}{\beta })\xi v''' = Re[2f'v' - vf''] + (M+K_p)v' > 0 \end{aligned}$$

It becomes contrary and hence $v'$ cannot have a maxima. So $v' = \frac{\partial f'}{\partial \alpha } > 0$.

IF we let two solutions $$f'(\xi ;\epsilon ^*)$$ and $$f'(\xi ;\epsilon ^{**})$$ with $$\epsilon ^{**} > \epsilon ^*$$, and using Mean Value Theorem

18 $$\begin{aligned} f'(\xi ;\epsilon ^{**}) - f'(\xi ;\epsilon ^*) = (\frac{\partial f'}{\partial \epsilon })_{\epsilon = {\hat{\epsilon }}}(\epsilon ^{**}- \epsilon ^*) = v'(\xi ;{\hat{\epsilon }})(\epsilon ^{**}- \epsilon ^*) \end{aligned}$$

where $$\epsilon ^*< {\hat{\epsilon }} < \epsilon ^{**}$$. Now $$v'$$ is bounded below by $$L > 0$$ for $$\xi $$ large as it cannot have a maximum. Suppose $$M = L(\epsilon ^{**}- \epsilon ^*)$$ and $$\xi \rightarrow \infty $$, From Eq. (18), $$0 = 1-1 = f'(\xi ;\epsilon ^{**}) - f'(\xi ;\epsilon ^*) = v'(\xi ;{\hat{\epsilon }})(\epsilon ^{**}- \epsilon ^*)> M > 0 $$

It becomes contrary.

It is to mentioned that the bounds for skin friction factor are evaluated and presented in the next part. $\square$

### Bounds for skin friction factor

Bounds are derived for coefficient of skin friction $f''(1) = \epsilon ^*$. As $f'(\xi ;\epsilon ^*)$ be a solution of the BVP to satisfy $f''(1;\epsilon ^*) = \epsilon ^* < 0$ and cannot have a maximum. It is claimed that for a solution to company the boundary condition (8), yields

19 $$\begin{aligned} f'''(1) =\frac{1}{(1+\frac{1}{\beta })}[ Re - \epsilon (1+Re\gamma ) + (M+K_p)] > 0 \end{aligned}$$

Consider,

Case-1: Solutions with $f'(\xi ;\epsilon ^*) > 0$ for $\xi > 1$: let $f'''(1) < 0$ as $f'$ is down concave initially. To satisfy Eq. (8), $f'$ must change concavity at some point. For some $\xi _3$ such that $f'(\xi _3) > 0$, $f''(\xi _3) < 0$, and $f'''(\xi _3) = 0$ with $f^{iv}(\xi _3) \ge 0$. Differentiating Eq. (5), yields:

20 $$\begin{aligned} (1+\frac{1}{\beta })\xi f^{iv} + (2+\frac{1}{\beta }+Ref)f''' - Ref'f''-(M+K_p)f'' = 0, \ 1< \xi < \infty , \end{aligned}$$

From Eq. (20), at $\xi = \xi _3$

21 $$\begin{aligned} (1+\frac{1}{\beta })\xi _3 f^{iv}(\xi _3) = Re f'(\xi _3)f''(\xi _3) + (M+K_p)f''(\xi _3) < 0 \end{aligned}$$

Also, seen in Lemma 1, $f'''(\xi _3) = f''(\xi _3) = 0$, so it becomes contrary. Next let $f'''(1) = 0$, in Eq. (20) to get:

22 $$\begin{aligned} f^{iv}(1) = \frac{1}{(1+\frac{1}{\beta })} [Re + (M+K_p)] \epsilon < 0 \end{aligned}$$

Then initially, $f''' < 0$ for $\xi > 1$, and $f'''$ cannot change sign.

Case-2: Solution for which $f'(\xi ;\epsilon ^*) < 0$: let $f'''(1) < 0$ and $f'$ is down concave initially. Because there exist a first point $\xi _4$ such that $f'(\xi _4) = 0$ and $f''(\xi _4) < 0$, $f'$ is not positive for all $\xi$. Also, $f'$ should be concave up to satisfy Eq. (8) for some $\xi > \xi _4$ and it attained a minimum. As $f'$ does not attain maximum, $f'$ necessarily increase from its minimum monotonically, and then tends to 0 from below to become concave down.

It becomes clear that, $f'''$ must change sign from minus to plus and back to minus. Thus a point $\xi _5$ is such that $f'''$ has a positive max, i.e., $f'''(\xi _5) > 0$, $f^{iv}(\xi _5) = 0$, and $f^{(v)}(\xi _5) \le 0$. The Eq. (20) is differentiated and evaluated at $\xi _5$ to produce,

23 $$\begin{aligned} \xi _5 f^{(v)}(\xi _5) = \frac{1}{(1+\frac{1}{\beta })} Re (f''(\xi _5))^2 \ge 0 \end{aligned}$$

If $f''(\xi _5) \ne 0$, contradiction is arrived: Taking the case $f''(\xi _5) = f^v(\xi _5) = 0$. The Eq. (20) is differentiated two times to have $f^{vi}(\xi _5) = 0$. Then Eq. (20) is differentiated thrice to get a result for $\xi = \xi _5$

$$\begin{aligned} \xi _5 f^{(vii)}(\xi _5) = \frac{1}{(1+\frac{1}{\beta })} 2 Re (f'''(\xi _5))^2 > 0. \end{aligned}$$

So finally, $f^{iv}(\xi _5) = f^v(\xi _5) = f^{vi}(\xi _5) = 0$ with $f^{vii}(\xi _5) > 0$. For $\xi$ nearly greater than $\xi _5$, $f^{iv}$ is positive and $f'''$ is increasing to contradict if $f'''$ is to possess maximum at $\xi _5$. We have

24 $$\begin{aligned} f'''(1) =\frac{1}{(1+\frac{1}{\beta })}[ Re - \epsilon ^* (1+Re\gamma ) + (M+K_p)] > 0 \end{aligned}$$

This bounds provides useful information, if $\gamma \ge -\frac{1}{Re}$. However, we have

25 $$\begin{aligned} \frac{Re+(M+K_p)}{1+Re\gamma }< \epsilon ^*,\ if\ \gamma < -\frac{1}{Re} \end{aligned}$$

then an upper bound on $\epsilon ^*$ can be attained if $\gamma \le -\frac{2}{R}$. At this stage , it is assumed that

26 $$\begin{aligned} f^{iv}(1) = \frac{1}{(1+\frac{1}{\beta })} (Re+M+K_p) \epsilon -\frac{1}{(1+\frac{1}{\beta })}(2+\frac{1}{\beta }+Re\gamma )[Re-(1+Re\gamma )\epsilon +(M+K_p)] < 0 \end{aligned}$$

First if $f^{iv}(1)>0$, then there exists a first point $\xi _6$ such that $f^{vi}(\xi _6) = 0$ with $f^v(\xi _6) \le 0$; otherwise,

27 $$\begin{aligned} f^{iv}(\xi )> 0,\ for\ \xi > 1 \end{aligned}$$

It will leads to a contradiction. Integration of Eq. (27) yields:

28 $$\begin{aligned} f'''(\xi )> K,\ for\ \xi > 1 \end{aligned}$$

where $K = \frac{1}{(1+\frac{1}{\beta })}[ Re - \epsilon (1+Re\gamma ) + (M+K_p)] > 0$. Integrating second time

29 $$\begin{aligned} f''(\xi )> \epsilon + K(\xi -1),\ for\ \xi > 1 \end{aligned}$$

When $\xi \rightarrow \infty$, let $f'' \rightarrow \infty$ then $f' \rightarrow 0$ as needed for Eq. (8).

**Table 1:** Values of $f''(1)$ for $Re = 1, M = K_p = 0.1\ and\ \beta = 10$.

$\gamma$	Lower bounds on $f''(1)$	$f''(1)$ num. approx	Upper bounds on $f''(1)$
− 0.5	NA	− 1.0007	NA
− 1.0	NA	− 0.8389	NA
− 3.0	− 0.600	− 0.4432	− 0.402
− 4.0	− 0.400	− 0.3414	− 0.340
− 6.0	− 0.240	− 0.2260	− 0.220
− 8.0	− 0.170	− 0.1663	− 0.160
− 10.0	− 0.133	− 0.1320	− 0.131

Thus $f^{iv}$ goes to decrease from 0 at some point $\xi _6$. Differentiate Eq. (20) and evaluate at $\xi _6$ to get

$$\begin{aligned} \xi _6 f^{(v)}(\xi _6) = Re (f''(\xi _6))^2 \ge 0 \end{aligned}$$

If $f''(\xi _6) \ne 0$, it makes a contradiction. If $f''(\xi _6) = 0$, then a similar procedure as above provides $f^{vi}(\xi _6) = 0$ and $f^{vii}(\xi _6) > 0$. Thus $f^{iv}>0$ for right interval of $\xi _6$, it is not negative as needed, so $f^{iv}(1) \not > 0$.

If $f^{iv}(1) = 0$ then Eq. (24) becomes $f^v(1) = R \epsilon ^2 > 0$ then contradiction is attained through above arguments. Solving for $\epsilon$ in Eq. (28) and using Eq. (25) yields.

30 $$\begin{aligned} \frac{Re+(M+K_p)}{1+Re\gamma }< \epsilon ^* < \frac{(Re+M+K_p)(2+\frac{1}{\beta }+Re\gamma )}{(1+\frac{1}{\beta })(R-M-K_p)+(2+\frac{1}{\beta }+R\gamma )(1+R\gamma )},\ if\ \gamma \le -\frac{2}{R} \end{aligned}$$

It can be noticed that both bounds converge to zero, and so, $f''(1)$ converges to zero as $\gamma \ (\gamma < 0)$ tends to infinity. Computations of skin friction coefficient $f''(1) = \epsilon ^*$ are provided in Table 1. Here sharpening of the bounds on $f''(1)$ is elucidated for a fixed a $Re = 1$, as the parameter $\gamma$ enhances.

The bound are acceptable for the solutions of the BVP if $\gamma \le -\frac{2}{R}$. Now we discuss the bounds for $\gamma > -\frac{1}{2R}$. firstly for $f'(\xi ;\epsilon ^*) > 0$ when $\xi > 1$, and secondly for $f'(\xi ;\epsilon ^* < 0)$. A lemma is presented for proof of Theorem 3.

#### Lemma 3

*Suppose* $f'(\xi ;\epsilon ^*) > 0$ *is solution of Eq.* (5) *with associated conditions *(8). *If* $\gamma > -\frac{1}{2Re}$, *then*

$$\begin{aligned} \lim _{\xi \rightarrow \infty }[- \xi (f''(\xi ))^2 + \frac{2Re}{3}(f'(\xi ))^3 + (M+K_p)(f'(\xi ))^2] = 0. \end{aligned}$$

#### Proof

From theorem 1 we take $f'(\xi ;\epsilon ^*) > 0$ for $\xi > 1$ and $f''(\xi ;\epsilon ^*) < 0$ for $\xi > 1$. Then f is increasing and $f'$ is decreasing function. As $\gamma > -\frac{1}{2Re}$, then $1-\frac{1}{\beta }+2Re\gamma > 0$ for $\xi > 1$. Multiplication of Eq. (5) with $f''(\xi )$ and integrating to get

31 $$\begin{aligned} {\left. \begin{aligned} \int _{1}^{\xi }(1-\frac{1}{\beta }+2Ref(z))(f''(z))^2 dz - (1+\frac{1}{\beta }) \epsilon ^2 + \frac{2Re}{3} + (M+K_p) = \ (1+\frac{1}{\beta })\xi (f''(\xi ))^2 + \frac{2Re}{3}(f'(\xi ))^3 + (M+K_p)(f'(\xi ))^2 \end{aligned}\right\} } \end{aligned}$$

Here, the LHS of equation Eq. (31) is an increasing function and similarly the RHS. As $f'(\xi ;\epsilon ^*)$ is a solution to the B.V.P, we have $f' \rightarrow 0$ as $\xi \rightarrow \infty$. As $- (1+\frac{1}{\beta })\xi (f''(\xi ))^2$ increases and bounded above by 0, its limit as $\xi \rightarrow \infty$ exists.

Also let limit is $l \ne 0$. Since $\lim _{\xi \rightarrow \infty } f'(\xi ) = 0$ and $-(1+\frac{1}{\beta })\xi (f''(\xi ))^2 < 0$ for $\xi > 1$, we must have $l < 0$. Suppose $l = -m$. Keeping in view That RHS of Eq. (31) is increasing, we have

$$\begin{aligned} - (1+\frac{1}{\beta })\xi (f''(\xi ))^2 + \frac{2R}{3}(f'(\xi ))^3 + (M+K_p)(f'(\xi ))^2 < -m \ for\ \xi \ge 1 \end{aligned}$$

and by skipping second term on LHS to get:

$$\begin{aligned} (1+\frac{1}{\beta })\xi (f''(\xi ))^2 > m \ for\ \xi \ge 1. \end{aligned}$$

It implies as:

$$\begin{aligned} (f''(\xi ) - \sqrt{\frac{m}{(1+\frac{1}{\beta })\xi }})(f''(\xi ) + \sqrt{\frac{m}{(1+\frac{1}{\beta })\xi }}) > 0 \ for\ \xi \ge 1, \end{aligned}$$

As the second term on the left is negative,

$$\begin{aligned} f''(\xi ) < \sqrt{\frac{m}{(1+\frac{1}{\beta })\xi }} \ for\ \xi \ge 1. \end{aligned}$$

Integration of this inequality provides as:

$$\begin{aligned} f'(\xi ) < 1 - 2 \sqrt{\frac{m}{(1+\frac{1}{\beta })}}(\sqrt{\xi }-1) \ for\ \xi \ge 1, \end{aligned}$$

and let $\xi \rightarrow \infty$ then $f' \rightarrow -\infty$ which is contradiction to Eq. (8). $\square$

#### Theorem 3

*Let* $f'(\xi ;\epsilon ^*) > 0$ *is a solution of Eq.* (5)*associated with boundary conditions* (8).*If* $\gamma > -\frac{1}{2R}$, *then* $\epsilon ^* < - \sqrt{\frac{1}{(1+\frac{1}{\beta })}[\frac{2Re}{3}+(M+K_p)]}$

#### Proof

Using Lemma 3 results and letting $\xi \rightarrow \infty$ in Eq. (31)

$$\begin{aligned} \int _{1}^{\xi }(1-\frac{1}{\beta }+2Ref(z))(f''(z))^2 dz = (1+\frac{1}{\beta })\epsilon ^2 - \frac{2Re}{3} + (M+K_p) > 0, \end{aligned}$$

since $1-\frac{1}{\beta }+2Rf > 0$ for $\xi > 1$. Thus $\epsilon ^* < - \sqrt{\frac{1}{(1+\frac{1}{\beta })}[\frac{2Re}{3}+(M+K_p)]}$.

Although the existence of solutions where $f'(\xi ;\epsilon ^*) < 0$ is yet an open problem. Suppose such solution exist, then a bound on the skin friction coefficient is established in next Theorem 4. Two lemmas are required for the proof of this bounds.

#### Lemma 4

*suppose there exist a solution of Eq.* (5) *associated with boundary conditions* (8) *where* $f'(\xi ;\epsilon ^*) < 0$. *Then* $\lim _{\xi \rightarrow \infty }(1+\frac{1}{\beta })\xi f''(\xi ) = 0$.

#### Proof

In preview of the case $\gamma \le -\frac{2}{Re}$, $f'$ must attain a negative minimum and then turn concave down as $f' \rightarrow 0$ from below. Thus there exist a point $\xi _7$ such that $f' < 0$, $f'' > 0$, and $f''' < 0$ for $\xi > \xi _7$. By using these inequalities and rearranging Eq. (5) into the form

32 $$\begin{aligned} (1+\frac{1}{\beta })\xi f''' + (1+Ref)f'' - Re(f')^2 -(M+K_p)f'= 0, \ 1< \xi < \infty , \end{aligned}$$

It is concluded that $f(\xi ) > -\frac{1}{Re}$ for $\xi > \xi _7$.

Hence f is decreasing and bounded below for $\xi > \xi _7$, and so, $f(\infty ) = l \ge -\frac{1}{Re}$ where *l* is finite. This results in

33 $$\begin{aligned} \lim _{\xi \rightarrow \infty } Re f(\xi ) f'(\xi ) = 0. \end{aligned}$$

Hence for all $\epsilon _1 > 0$, there is ${{\bar{\xi }}} > \xi _7$ to yield:

34 $$\begin{aligned} -\frac{\epsilon _1}{4}< Re f(\xi ) f'(\xi ) < \frac{\epsilon _1}{4} \ for \ \xi >{{\bar{\xi }}}_7. \end{aligned}$$

Keeping in view of contradiction, suppose that $\lim _{\xi \rightarrow \infty } (1+\frac{1}{\beta })\xi f''(\xi ) \ne 0$, there exist an $\epsilon _1 > 0$ and a sequence $\xi _i \rightarrow \infty$ such that $|(1+\frac{1}{\beta })\xi _i f''(\xi _i)| \ge \epsilon _1$ for $i = 1, 2, .....$ and since $f'' > 0$ for $\xi >{{\bar{\xi }}}_7$, we have

35 $$\begin{aligned} (1+\frac{1}{\beta })\xi _i f''(\xi _i) \ge \epsilon _1 \ for \ \xi _i > \xi _7. \end{aligned}$$

For any positive integer N, the inequalities (34), (37) hold where $\xi _N> {{\bar{\xi }}} > \xi _7.$ We get

36 $$\begin{aligned} (1+\frac{1}{\beta })\xi _i f''(\xi _i) + Re f(\xi _i) f'(\xi _i) > \epsilon _1 -\frac{\epsilon _1}{4} = \frac{3\epsilon _1}{4} \ for \ \xi _i \ge \xi _N. \end{aligned}$$

Arrangements of Eq. (13) yields

37 $$\begin{aligned} 2Re\int _{1}^{\xi }(f'(z))^2 dz + Re \gamma + (1+\frac{1}{\beta })\epsilon = (1+\frac{1}{\beta })\xi f''(\xi ) + Re f(\xi ) f'(\xi ) + Re[(M+K_p)(f(\xi )-\gamma )], \end{aligned}$$

here LHS is increasing. It is concluded that the inequality (36) stands for all $\xi \ge \xi _N$ and (36) becomes

38 $$\begin{aligned} (1+\frac{1}{\beta })\xi f''(\xi ) \ge \frac{3\epsilon _1}{4} - Re f(\xi ) f'(\xi ) \ for \ \xi \ge \xi _N \end{aligned}$$

and using (34) in (38) yields $$\square $$$$(1+\frac{1}{\beta })\xi f''(\xi ) \ge \frac{\epsilon _1}{2}$$ for $$\xi \ge \xi _N$$.

Dividing both sides by $\xi$ and integrating results in $f'(\xi ) \ge f'(\xi _N) + \frac{\epsilon _1}{2}[ln \xi - ln \xi _N]$ for $\xi \ge \xi _N$.

Finally, suppose $\xi \rightarrow \infty$ and $f' \rightarrow \infty$ which contradict Eq. (8) and thus proof of lemma is complete.

#### Lemma 5

*Let there exists a solution of Eq.* (5) *with boundary conditions *(8) *when* $f'(\xi ;\epsilon ^*) < 0$*provided that* $\gamma > -\frac{1}{2R}$, $\int _{1}^{\infty }(1-\frac{1}{\beta }+2Rf(z))(f''(z))^2 dz > 0.$

#### Proof

It is sufficient to show that $1-\frac{1}{\beta }+2Rf > 0$ for $\xi \ge 1$. From Lemma 4,it is seen that $f' < 0$, $f'' > 0$ and $f''' < 0$ for $\xi > \xi _7$. Hence $f'' > 0$ and decreasing and $f'(\infty )$ exists, then $f''(\infty ) = 0$. Suppose $\xi \rightarrow \infty$ in Eq. (31) and using Lemma 4 to get

$$\begin{aligned} \lim _{\xi \rightarrow \infty }[-\xi (f''(\xi ))^2 + \frac{2Re}{3}(f'(\xi ))^3 + (M+K_p)(f'(\xi ))^2] = 0, \end{aligned}$$

and thus

39 $$\begin{aligned} \int _{1}^{\infty }(1-\frac{1}{\beta }+2Ref(z))(f''(z))^2 dz = (1+\frac{1}{\beta })\epsilon ^2 - \frac{2Re}{3}+(M+K_p). \end{aligned}$$

Also, we have

40 $$\begin{aligned} {\left. \begin{aligned} \int _{1}^{\xi }(1-\frac{1}{\beta }+2Rf(z))(f''(z))^2 dz - (1+\frac{1}{\beta })\epsilon ^2 + \frac{2Re}{3}+(M+K_p) =\\ -(1+\frac{1}{\beta })\xi (f''(\xi ))^2 + (M+K_p)(f'(\xi ))^2 + \frac{2R}{3}(f'(\xi ))^3 < 0 \ for \ \xi > \xi _7 \end{aligned}\right\} } \end{aligned}$$

It is to note that both terms on the right are negative, and so,

41 $$\begin{aligned} \int _{1}^{\xi }(1-\frac{1}{\beta }+2Ref(z))(f''(z))^2 dz= (1+\frac{1}{\beta })\epsilon ^2 - \frac{2Re}{3} ++(M+K_p) \ for \ \xi > \xi _7. \end{aligned}$$

Thus $\int _{1}^{\xi }(1-\frac{1}{\beta }+2Ref(z))(f''(z))^2 dt$ tends to infinity from below, and $1-\frac{1}{\beta }+2Ref$ is to be positive for large values of $\xi$. Since $\gamma > -\frac{1}{2Re}$, and $1-\frac{1}{\beta }+2Ref$ starts out positive because $f'$ has only one sign change − from positive to negative $-f$ attains one maximum and so does $1-\frac{1}{\beta }+2Ref$. Thus $1-\frac{1}{\beta }+2Ref > 0$ for $\xi \ge 1$ and hence, the proof of lemma.

#### Theorem 4

*Let there is a solution for Eq.* (5) *associated with the boundary conditions* (8) *where* $f'(\xi ;\epsilon ^*) < 0$. *If* $\gamma > -\frac{1}{2Re}$, *then* $\epsilon ^* < min [{- \sqrt{\frac{1}{(1+\frac{1}{\beta })}[\frac{2Re}{3}+(M+K_p)]}, -\frac{Re\gamma }{(1+\frac{1}{\beta })}}].$

#### Proof

Suppose $\xi \rightarrow \infty$, using Lemma 4, Eq. (33) in Eq. (37) to achieve as below

42 $$ \begin{aligned}    & \int_{1}^{\infty } {(f^{\prime\prime}(} z))^{2} dz = \left( {\frac{{\left( {1 + \frac{1}{\beta }} \right)\epsilon  + Re\gamma }}{{2Re}}} \right) > 0, \\     & \epsilon ^{*}  <  - \frac{{Re\gamma }}{{\left( {1 + \frac{1}{\beta }} \right)}} \\  \end{aligned}  $$

Using Lemma 5 in Eq. (41) to get

43 $$\begin{aligned} \epsilon ^* < - \sqrt{\frac{1}{(1+\frac{1}{\beta })}[\frac{2Re}{3}+(M+K_p)]}, \ if \ \gamma > -\frac{1}{2Re}, \end{aligned}$$

From combining the inequalities (42), (43), we get $\epsilon ^* < min [{- \sqrt{\frac{1}{(1+\frac{1}{\beta })}[\frac{2Re}{3}+(M+K_p)]}, - \frac{Re\gamma }{(1+\frac{1}{\beta })}}]$, if $\gamma > -\frac{1}{2Re}.$

$$\square $$

## Results and discussion

The current results are checked for validation as listed in Table 2 and 3. Their acceptable accord with those by Mastroberardino and Siddique^41^ has established the accuracy of the present numeric scheme. The pictorial representation for Casson nano-fluid’s velocity, temperature and concentration of nano-entities graphed for two cases of mass transpiration $(\gamma > 0, \gamma < 0)$.

**Table 2:** The skin friction coefficient by varying *M* and *Re*.

*M*	*Re*	$\gamma =0.5$^41^	$\gamma =0.5$ (Present results)	Percentage deviation	$\gamma =-\,0.5$^41^	$\gamma =-0.5$ (present results)	Percentage deviation
0	10	− 6.62227	− 6.6223	0.000453	− 1.67757	− 1.6778	− 0.013710
2		− 6.88470	− 6.8847	0.000000	− 1.92938	− 1.9294	0.001036
5	10	− 7.24505	− 7.2451	0.000690	− 2.27933	− 2.2793	− 0.001316
2	1	− 2.21659	− 2.2180	0.063611	− 1.72075	− 1.7214	0.037774
	5	− 4.33228	− 4.3330	0.016619	− 1.86364	− 1.8638	0.008585
	10	− 6.88470	− 6.8847	0.000000	− 1.92938	− 1.9294	0.001036

**Table 3:** Nusselt number table for varying $\gamma$, *Re*, *M*, and *Pr*.

$${{\gamma }}$$	$${Re} $$	*M*	*Pr*	Mastroberardino and Siddique^41^	Present results	Percentage deviation
0.5	10	2	7	36.60283	36.6027	− 0.0003551
0.0				6.08375	6.0857	0.0320525
− 0.5				0.00002	0.00002	0.0000000
0.5	1			4.57611	4.5741	− 0.0439237
	5			18.99556	18.9952	− 0.0018951
	10			36.60283	36.6027	− 0.0003551
	10	0		36.60105	36.6115	0.0285510
		2		36.60283	36.6027	− 0.0003551
		5		36.60551	36.5906	− 0.0407315
		2	0.7	4.18133	4.1801	− 0.294164
			2	11.13801	11.1360	− 0.0180463
			7	36.60283	36.6027	− 0.0003551

**Figure 2:**
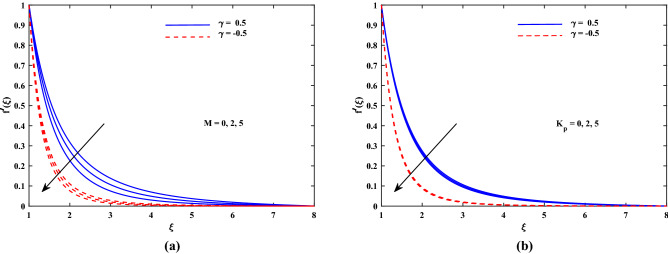
Plot for velocity profile $f'(\xi )$ with varying values of *M* and $K_p$.

**Figure 3:**
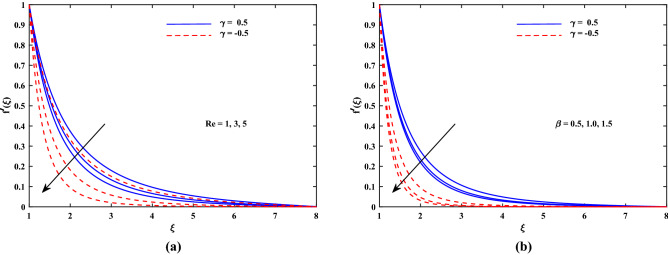
Plot for velocity profile $f'(\xi )$ with varying values of *Re* and $\beta$.

**Figure 4:**
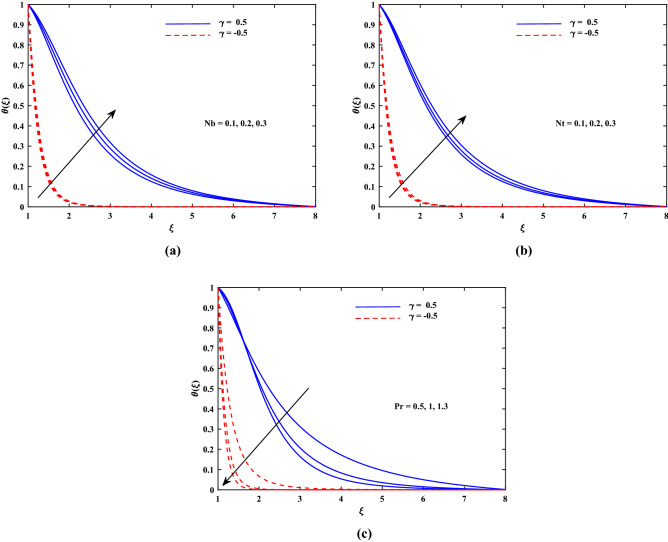
Plot for temperature profile $\theta (\xi )$ with varying values of *Nb*, *Nt* and *Pr*.

**Figure 5:**
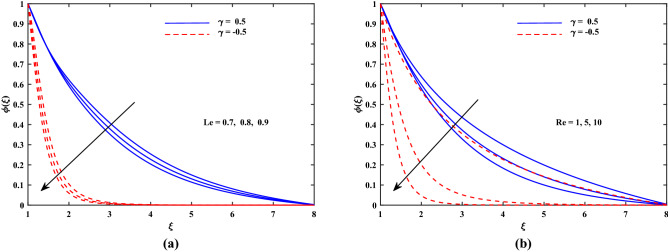
Plot for concentration profile $\phi (\xi )$ with varying values of *Le* and *Re*.

**Table 4:** Skin friction $-\,f''(0)$ for varying $K_p$, *M*, *Re*, and $\beta$.

$K_p$	*M*	*Re*	$\beta$	$\gamma = -\,0.5$	$\gamma = 0$	$\gamma = 0.5$
0.5	2	10	0.5	1.5608	2.2392	3.1970
1.0				1.6001	2.2818	3.2380
1.5				1.6386	2.3234	3.2781
0.5	0			1.3953	2.0576	3.0218
	2			1.5608	2.2392	3.1970
	5			1.7862	2.4814	3.4307
	2	1		1.2200	1.3002	1.3851
		5		1.4208	1.7861	2.2385
		10		1.5608	2.2392	3.1970
		10	0.5	1.5608	2.2392	3.1970
			1.0	1.7271	2.6988	4.1909
			1.5	1.8000	2.9378	4.7615

**Table 5:** Nusselt number $-\,\theta '(0)$ and Sharwood number $-\,\phi '(0)$ for varying *Pr*, *Nb*, *Nt*, *Le* and *Re* .

*Pr*	*Nb*	*Nt*	*Le*	*Re*	$-\,\theta '(0)$	$-\,\phi '(0)$
0.72	0.1	0.1	1	10	4.2980	
0.1					5.6546	
1.3					7.0521	
0.72	0.1				4.2980	
	0.2				4.1206	
	0.3				3.9488	
	0.1	0.1			4.2980	
		0.2			4.2028	
		0.3			4.1100	
		0.1	1			2.4484
			2			7.8881
			3			13.1081
			1	1		0.6743
				5		1.4877
				10		2.4484

The outcomes for velocity $f'(\xi )$, temperature $\theta '(\xi )$ and concentration $\phi '(\xi )$ are sketched in Figs. 2, 3, 4 and 5 for two cases of $\gamma (\gamma = -0.5 and \gamma = 0.5)$ with the variation of other influential parameters. The velocity $f'(\xi )$ is vividly decelerated against the increments in magnetic parameter *M* as well as that of porosity parameter $K_p$ as seen in Fig. 2. The strength of *M* means growth of electromagnetic resistive force (Lorentz force) which inhibits the flow. Similarly, parameter of porous matrix $(K_p)$ offers enhanced resistance to the velocity. There is sound reason behind this fact that $K_p$ is related reciprocally with permeability and hence higher inputs of $K_p$ means lesser permeability. Thus the flow decelerates in this case. The incremented values of *Re* and Casson parameter $\beta$ also slowed the flow velocity $f'(\xi )$ as delineated in Fig. 3. Here the viscous effects are enhanced (to oppose to) momentum. Furthermore, it is noticed that velocity of flow is faster in case of injection $(\gamma > 0)$ than for suction $(\gamma < 0)$. Figure 4 exposed that the nanofluid diffusion parameters namely *Nb* (Brownian diffusion) and *Nt* (Thermophoresis diffusion) are responsible to raise the temperature function $\theta (\xi )$ but the progressive values of *Pr* reduced $\theta (\xi )$. The faster random motion of nano-entities is associated with larger *Nb*. This rapidity in the movement of these small material particles causes greater thermal distribution. In the similar behavior due to enhanced enhanced thermophoresis. The particles move fastly towards cooler regimes and hence raises the temperature. It is also noticed that the fluid temperature for suction is higher than for injection. The greater values of *Le* and *Re* diminish the nanoparticle concentration $\phi (\xi )$ in the boundary layer region as depicted in Fig. 5. Physically, the Lewis number *Le* is inversely related to diffusion coefficient of concentration and hence its development impairs $\phi (\xi )$. Moreover as seen above, the larger *Re* slows the flow which results in decrement of $\phi (\xi )$

The absolute values of skin friction are augmented in direct proportion with $K_p$, *M*, *Re* and $\beta$ for three cases of $\gamma$ $(\gamma < 0, \gamma = 0, \gamma > 0)$ as enumerated in Table 4. Physically, $K_p$ signifies the resistance of porous matrix, *M* for electromagnetic resistive force, *Re* (Reynolds number) and $\beta$ the non-Newtonian viscous effects (for Casson fluid). Hence the drag force enhances. Table 5 indicates that Nusselt number $-\theta '(0)$ increases with *Pr* but it diminishes against *Nb* and *Nt*. Physical ground for augmentation of Nusselt number with Prandtl number lies in the fact that thermal diffusivity being reciprocal to prandtl number is responsible to decrease the temperature of the fluid and more heat transfer takes place at the surface and hence the magnitude of Nusselt number is boosted. Further the growing thermopherotic and Brownian diffusion raise the fluid temperature and heat transfer rate at the surface is decreased and the Nusselt number decreases against these parameters. Also, the Sherwood number $-\phi '(0)$ exceeds directly with *Le* and *Re* (see Table 5).

## Conclusion

We discussed the existence of solution for Casson fluid flow towards a porous stretching cylinder. The fluid flows through porous medium and it is influenced by magnetic field. It is shown that the boundary value problem for any $Re > 0$ and $-\infty< \gamma < \infty$, to satisfy $f'(\xi ) > 0$ and $f''(\xi ) < 0$ for all $\xi > 1$. The uniqueness of the result is established in the sense that we cannot have two solutions for the boundary value problem if $-\infty< \gamma < \infty$ and $Re > 0$. Moreover, the bounds for skin friction factor are evaluated. Numerical solution of the flow and heat transfer for Casson nano-fluid is also obtain to reveal that:

- It is observed that for the magnetic parameter *M* and porosity parameter *kp* reduces velocity when takes large values for three cases of $\gamma$.
- Velocity recedes with the higher inputs of *Re* and $\beta$.
- Temperature decreases with the boosting values of *Pr* but it uplifted with higher values of *Nb* and *Nt*.
- *Le* and *Re* cause deprecation in concentration when takes larger values.
- Skin friction factor is boosted up significantly when takes larger $K_p$, *M*, *Re* and $\beta$.
- Nusselt number and Sherwood number up surged directly with larger *Pr*, *Le* and *Re* while recedes for larger *Nb* and *Nt*.

## Nomenclature

*u*: Radial velocity component
*w*: Axial velocity component
*a*: Radius of cylinder
*c*: Strain rate of oncoming radial flow
*Nb*: Brownian motion
$D_B$: Brownian diffusion coefficient
*Le*: Lewis number
*T*: Non-dimensional temperature
*C*: Non-dimensional nanoparticles concentration
$C_w$: Nanoparticles concentration at surface
$C_\infty$: Nanoparticles concentration away from the surface
$$Nu_{x}$$: Nusselt number
*Pr*: Prandtl number
$$C_{fx}$$: skin friction
$$Sh_x$$: Sherwood number
*Nt*: Thermophoresis parameter
$D_T$: Thermophoretic diffusion coefficient
$T_w$: Temperature at surface
$T_{\infty }$: Temperature away from the surface
*Re*: Reynolds number
*M*: Magnetic field
$K_p$: Permeability of porous medium
$U_w$: Velocity of stretching sheet.

## Greek symbols

$\rho$: Density of fluid
$$\nu $$: Kinematic viscosity
$\tau$: Ratio between hear capacities of the fluid and nanoparticles
$$\mu $$: Dynamic viscosity
$$\gamma $$: Permeability parameter
$$\alpha $$: Thermal diffusivity
$$\theta $$: Dimensionless temperature
$$\nu $$: Kinematic viscosity
$$\beta $$: Casson parameter
$\Phi$: Nanoparticles volume fraction.
